# Virility, pleasure and female genital mutilation/cutting. A qualitative study of perceptions and experiences of medicalized defibulation among Somali and Sudanese migrants in Norway

**DOI:** 10.1186/s12978-017-0287-4

**Published:** 2017-02-10

**Authors:** R. Elise B. Johansen

**Affiliations:** Norwegian Center for Violence and Traumatic Stress Studies, NKVTS, PB: 181 Nydalen, 0409 Oslo, Norway

**Keywords:** Infibulation, Defibulation, Migration, Change, Female genital mutilation/cutting

## Abstract

**Background:**

The most pervasive form of female genital mutilation/cutting—infibulation—involves the almost complete closure of the vaginal orifice by cutting and closing the labia to create a skin seal. A small opening remains for the passage of urine and menstrual blood. This physical closure has to be re-opened—defibulated—later in life. When they marry, a partial opening is made to enable sexual intercourse. The husband commonly uses his penis to create this opening. In some settings, a circumciser or traditional midwife opens the infibulated scar with a knife or razor blade. Later, during childbirth, a further opening is necessary to make room for the child’s passage. In Norway, public health services provide surgical defibulation, which is less risky and painful than traditional forms of defibulation.

This paper explores the perceptions and experiences of surgical defibulation among migrants in Norway and investigates whether surgical defibulation is an accepted medicalization of a traditional procedure or instead challenges the cultural underpinnings of infibulation.

**Methods:**

Data derived from in-depth interviews with 36 women and men of Somali and Sudanese origin and with 30 service providers, as well as participant observations in various settings from 2014–15, were thematically analyzed.

**Results:**

The study findings indicate that, despite negative attitudes towards infibulation, its cultural meaning in relation to virility and sexual pleasure constitutes a barrier to the acceptance of medicalized defibulation.

**Conclusions:**

As sexual concerns regarding virility and male sexual pleasure constitute a barrier to the uptake of medicalized defibulation, health care providers need to address sexual concerns when discussing treatment for complications in infibulated women. Furthermore, campaigns and counselling against this practice also need to tackle these sexual concerns.

## Plain English summary

The most pervasive form of female genital mutilation/cutting—infibulation—involves the almost complete closure of the vaginal orifice by cutting and closing the labia to create a skin seal. A small opening remains for the passage of urine and menstrual blood. Upon marriage and childbirth, this closure needs to be opened—i.e., defibulated. After marrying, the husband traditionally uses his penis or a circumciser uses a knife or razor blade to open this seal sufficiently for sexual intercourse. In Norway, public health services provide surgical defibulation, which is performed to reduce the pain and risks involved in traditional forms of defibulation and to reduce birth complications.

This paper explores how Somali and Sudanese migrants in Norway relate to medicalized defibulation offerings. It also investigates whether surgical defibulation is an accepted medicalization of a traditional procedure or instead challenges the cultural underpinnings of infibulation. A qualitative study, including in-depth interviews with 36 women and men of Somali and Sudanese origin and 30 service providers, as well as participant observations, was conducted from 2014–15. The study found that, while informants had negative attitudes toward infibulation, many of the associated cultural values were still upheld and constituted a barrier to the uptake of medicalized defibulation. Medicalized defibulation was seen to undermine male virility and masculinity, which was expected to be expressed through penile defibulation. Furthermore, medicalized defibulation was considered a threat to the tight vaginal opening that was regarded as a prerequisite for male sexual pleasure.

## Background

Medicalized defibulation is a surgical procedure constituting a partial undoing of infibulation—the most extreme form of female genital mutilation/cutting (FGM/C) [1]. Discourses and practices relating to this procedure’s acceptance and uptake are used as an empirical entry for studying the continuity and changes in the cultural meaning of infibulation. The study’s context concerns Somali and Sudanese migrants living in Norway.

In Somalia and the Democratic Republic of Sudan, infibulation is nearly universally practiced and is associated with a complex set of key cultural values. These values hinge on ideals and practices related to women’s virginity and virtue and men’s virility and sexual pleasure [2–4]. Despite these cultural values, the United Nations define FGM/C as a violation of human rights [1] because of the health risks associated with the practice and because it is almost exclusively performed on minors [1, 5, 6]. Therefore, in recent decades, numerous interventions have arisen to promote its abandonment [7, 8]. However, while support for the practice is decreasing, the decrease in the practice itself is less pronounced [9]. This discrepancy between attitudes and practices might reveal a resistance to change that has been underestimated and, in turn, has not been appropriately addressed. More pervasive changes in the support for FGM/C have been identified in diaspora communities, particularly against infibulation [10–13], and this study explores the practical implications with regard to the acceptance of defibulation.

Studies on attitudes toward the practice of FGM/C often suffer from Methodological limitations. While studies ask whether people have negative or positive attitudes toward the practice [11], research has shown attitudes to be both complex and fluid [14–17]. Furthermore, several studies have found that individuals with negative attitudes toward FGM/C may be unable to put their conviction into practice due to social pressures [14, 18]. In recent research on FGM/C, the interdependence between individual conviction and social norms has been a major motivation for a strong focus on social norms [9]. Central to these studies are Garry Mackie’s efforts to explain why people continue following a social convention that they no longer support [19]. Mackie’s theories suggest that people continue practicing FGM/C mainly because everyone else does; consequently, this practice has become a prerequisite for marriage. Therefore, the key to abandoning this practice involves establishing a joint agreement to do so; the social convention will thereby be broken, and the underlying social norms will dissolve. However, this paper suggests that change must go deeper and that negative attitudes toward FGM/C must translate into profound changes in the underlying cultural values [20, 21]. Therefore, this study explores a new avenue for understanding cultural change. It relies on the utilization of medicalized defibulation for those already subjected to the practice rather than on stated attitudes towards the practice or data on its prevalence.

Medicalized defibulation reduces the suffering and risk associated with traditional forms of defibulation. Therefore, given the widespread negative attitudes toward infibulation in the diaspora, girls and women subjected to pre-migration infibulation could be expected to eagerly embrace access to clinical defibulation in Norway. That is, if infibulation is no longer of significant importance, no cultural convention should require that women refrain from clinical defibulation. In contrast, people’s resistance to surgical defibulation could imply that some cultural underpinnings of infibulation are still significant in the community.

### Female genital mutilation/cutting among Somali and Sudanese populations

Population-based prevalence data from 30 countries estimate that approximately 200 million girls and women have undergone FGM/C [22]. The practice is particularly widespread in Somalia and the Democratic Republic of Sudan, with occurrence rates of 98 and 99% in the two Somali states of Somaliland and Puntland, respectively [23, 24], and 87% in Sudan [25]. Through migration, the practice is now found worldwide. In Norway, approximately 17,300 girls and women are estimated to have undergone FGM/C prior to immigration [26]. Half are of Somali origin, and approximately 3% are of Sudanese origin [26]. Together, they constitute a major proportion of girls and women who have undergone the most pervasive type of FGM/C in Norway.

FGM/C is a general term covering a variety of procedures, which are classified into four major types by the World Health Organization (WHO): Type I – removal of part or all of the clitoris; Type II – removal of part or all labia minora and often the clitoris; and Type III – cutting and apposition of the labia, creating a seal of skin that closes the vulva and most of the vaginal opening [1]. This study focuses on Type III, commonly referred to as infibulation. Type IV comprises any other procedures that can harm the external genitalia but that do not include tissue removal.

In Somalia and Sudan, the emic classification outlines two major types of FGM/C: “*pharaonic*” and “*sunna*”. “*Pharaonic”* refers to Type III FGM/C, highlighting a common belief that the practice originated in Egypt. Infibulation is the predominant form of FGM/C in both countries, with occurrence rates of 87% in Somaliland [23], 85% in Puntland [24] and 82% in Sudan [27]. Approximately 9,100 girls and women in Norway have been estimated to have undergone pre-migration infibulation [26]. However, the actual prevalence of infibulation is likely even higher, as the extent of FGM/C is generally underreported [28–31]. Underreporting partly results from the lack of a uniform definition regarding what constitutes “sunna” as well as clinical evidence suggesting that many women who claim to have sunna FGM/C are infibulated [17]. “Sunna” is generally described as less extensive and harmful than infibulation, often as a “minor cut”, but in practice the term is used to refer to any of the four types [30, 32, 33].

Infibulation constitutes a densely meaningful symbol that is intrinsically intertwined with the physiological extent of the procedure. The opening left in the infibulated scar should be sufficiently small to impede sexual intercourse to fulfill its major function of safeguarding and proving virginity [2–4, 34]. Nevertheless, this virtuous closure must later be reopened to fulfill cultural values related to marriage and motherhood. First, a partial opening is made at the time of marriage to enable sexual intercourse and conception. At the time of childbirth, a more substantial opening is needed to provide room for the passage of the baby.

These opening procedures are not only a technical necessity but also highly significant cultural, symbolical and personal experiences. Through defibulation, a girl is transformed from a single virginal girl to a mature woman—married and ready for motherhood. It also provides her husband with access to her sexual and reproductive powers and services [4, 35]. The traditional defibulation process, whereby the man opens his bride’s vaginal orifice with his penis, is further associated with his virility and strength, thus providing evidence of his masculinity [3, 4, 18]. Furthermore, a small, only partially open vaginal orifice is considered essential for male sexual pleasure and, in turn, fertility and marital stability [34].

### Traditional and medicalized defibulation

To understand whether and in what ways medicalized defibulation would involve cultural changes in terms of the meanings of FGM/C, the similarities and differences between traditional and medicalized defibulation needs to be outlined.

Traditional defibulation at the time of marriage is performed in one of two ways. First, in Sudan and southern Somalia, the bridegroom is expected to defibulate his bride through penile penetration [4, 34, 36]. To ensure a sufficient opening, the man is expected to put sufficient pressure on the infibulation seal, causing it to tear. This practice is painful for both women [35, 37–39] and men [3, 4, 18, 40]. Depending on various factors, including the amount of force used, the orifice’s size, and the seal’s thickness and scarring, the time required to defibulate varies, but it is generally expected to be accomplished within a week [35, 37]. Occasionally, men are said to use tools, such as knives or razor blades, if penile pressure proves insufficient [36]. In northern Somalia, an excisor (circumciser) is commonly called on to cut open the infibulation [2]. However, whether the opening is ensured through penile penetration or the use of a cutting tool, the couple have to engage in regular sexual intercourse during the following weeks to prevent the infibulation from healing, thus recreating infibulation and closing the vulva [35, 37]. This “maintenance” period is also painful, as sexual intercourse occurs despite the presence of open wounds, and infections and bleedings are common [35, 37]. Many women describe the defibulation procedure as equally painful as the original infibulation [18, 38].

In preparation for childbirth, a further opening is necessary to make room for the passage of the child. This opening is generally performed by a birth assistant, whether a traditional birth attendant or an educated midwife, who often has performed the original FGM/C. After childbirth, the cut edges are treated in different ways. In Sudan, reinfibulation, whereby the two sides of the labia are re-sutured, is a routine post-delivery procedure [41, 42]. This closure (*al-adil*) commonly goes beyond merely closing what was opened during delivery and includes cutting or scraping new tissue to recreate a vaginal orifice similar to that of an unmarried woman [3, 41, 42]. In such cases, a new process of defibulation for sexual intercourse is necessary, leading women to go through repeated closure and openings throughout their childbearing years [40–44]. Less is known about post-delivery care procedures in Somali. No clear evidence has shown that reinfibulation is common there, although one study from Kenya has suggested such practices [36].

To accommodate the health care needs of women with FGM/C, and particularly to reduce the risks of birth complications that affect both mother and child [45], Norwegian health care authorities have developed medical guidelines to encourage defibulation before pregnancy (preferably), during pregnancy, or during childbirth [46, 47]. They have also established eight specialized clinics across the country to address the needs of girls and women with FGM/C [48].

To ease access to these services, some clinics accept women who seek help directly. Others require referrals, which are easy to access and are accepted from various service providers. The cost is also low at approximately 34 Euro (NOK 320), as medicalized defibulation is offered as part of public health care services. Finally, travel time and cost is also low for most women, as the clinics are located in major cities with the highest concentrations of affected migrant groups [49].

Medicalized defibulation differs from traditional defibulation modes in several ways. First, medicalized defibulation is performed clinically, with pain relief and sterile instruments. The Norwegian guidelines advise sufficient defibulation to uncover the urethra [46]. This is expected to ease daily functioning of urination and menstruation and to facilitate eventual medical examinations and childbirth. The cut edges are sutured to each side to prevent regrowth and re-closure. Furthermore, couples are advised to refrain from sexual intercourse until the wounds heal.

Compared with traditional procedures, medicalized defibulation likely reduces pain, risk of infection, and other complications significantly. It also reduces the need for further defibulation when women give birth. If not done before, defibulation is a necessity in childbirth to avoid uncontrolled tearing, though occasionally health care providers have preferred to carry out multiple episiotomies instead, though they are more invasive procedures [18]. Given these benefits, infibulated women and their male partners can be expected to prefer medicalized defibulation over painful and time-consuming traditional practices.

However, no accurate data report an uptake of medicalized defibulation to support this assumed preference. A newspaper article reported that 127 women had sought help for FGM/C-problems in 2013 [50], but how many of these women underwent medicalized defibulation is unknown. Given that more than 9,100 women in Norway most likely have undergone infibulation, an underutilization of such services can be inferred. Does this limited uptake indicate a resistance to medicalized defibulation?

This study thus seeks to explore the factors that encourage and hinder women and girls from seeking medicalized defibulation. A deeper understanding of these factors can improve our understanding of health-seeking behavior, the utilization of medicalized defibulation and the acceptance of these services. The findings may also identify factors relevant to changes in the practice of FGM/C and help assess the readiness to change among those affected.

## Methods

A qualitative study, including interviews and participant observations in Somali and Sudanese communities was conducted in the period 2014–2015. Efforts were made to recruit informants from diverse backgrounds. Informants were recruited from across the country—approximately half from Oslo and the remainder from eight other towns and villages.

In-depth interviews with key informants were conducted with 23 women and 13 men of Somali and Sudanese origin. Twenty-two were of Somali origin, and 14 were of Sudanese origin. Twenty-eight of the interviewees were referred to as “settled” (14 Sudanese and 14 Somali), and they were recruited in two ways. Snow-ball sampling through different starting points was used to recruit 24 informants who had lived more than a year in Norway, and four key informants were recruited through the services in which they worked. In addition, eight newly arrived Somali quota refugees were included in the study. These refugees were recruited through the immigration authorities (“new” in Table 1).

**Table 1:** Overview of somali and sudanese informants for in-depth interviews

Gender	Total	Somalia	Sudan	Length of stay	Marital status
Women	23	14	9	New (6): 3–12 months	Single −2
				Settled (17): 3–30 years	Married −13
					Divorced/Widowed −8
Men	13	8	5	New (2): 3 months	Single 5
				Settled (11):10–34 years	Married 6
					Divorced/Widowed-2
Total	36	22	14	Average 15 years	
				(Sudanese 6 years, Somali 18 years)	

The recruitment strategies that were selected to include informants with various lengths of stay and migration routes thus resulted in two informant groups: long-term residents and newly arrived refugees. The contacts who assisted in the initial recruitment of settled informants had high levels of education and long-term residence in Norway. This bias was also evident among the informants who they recruited, of whom the majority had higher levels of education (beyond primary school) and employment than the average Somali and Sudanese migrants in Norway. This bias was particularly pronounced among the Sudanese, several of whom had studied at the university level, both in Sudan and Norway. The settled informants thus differed significantly from the average Somali and Sudanese migrant in sense of higher education and level of employment. By contrast, the newly arrived Somali refugees had no or minimal education and none was employed.

The informants’ ages ranged from 18 to 65, and most were in their 30s and 40s. No systematic age difference existed between the various subgroups (men, women, Somali, Sudanese, newly arrived refugees or settled informants). Somali informants came from all over Somalia, and one came from a neighboring country. The Sudanese informants originated from different regions within northern Sudan, though two had grown up in different neighboring countries.

Almost all the women had been subjected to FGM/C, except one Somali and one Sudanese woman. Of those with FGM/C, all but one was infibulated. Although three other women claimed to have *sunna,* their subsequent stories included experiences of closure and opening that indicated some extent of infibulation. One male informant said that his wife had no FGM/C, whereas the other men reported infibulated wives and ex-wives.

The 30 public servants were recruited through formal channels based on their experience and work with FGM/C and/or refugees. These recruits included employees from health clinics that conducted defibulation, school nurses, sexual counselors for youth, and personnel responsible for selecting, interviewing and providing information and medical care for refugees and asylum seekers.

Participant observations were conducted in various settings in which FGM/C was on the agenda. This included homogenous and mixed groups with regard to gender, nationality and age. In these and other settings, informal conversations were conducted with an additional 30–40 men and women. Though notes were taken when topics concerning this study were raised during these sessions and conversations, they are not directly referred in the paper. Rather they were used to double-check and as a sounding board for the findings from the interviews. Finally, two validation seminars with Somali and Sudanese men and women were conducted in two different cities. A draft analysis and a selection of quotation from interviews were presented for discussion at these seminars.

Interviews were conducted by the researcher, mostly in Norwegian or English, and lasted from 20 minutes to 4 hours. The newly arrived Somali refugees were interviewed with the assistance of a Somali-speaking co-interviewer. All Sudanese informants spoke either English or Norwegian, and they were interviewed by the researcher. The informants chose the venue for the interview, including informants’ homes, the researcher’s workplace, the informants’ workplaces, the refugee or social service office, or a public space, such as a coffee shop or a park.

The study was described to potential informants as follows: “Several hospitals in Norway offer help to women who have been circumcised. We will examine what people know about this, what they think and their experiences, why some seek help and others do not, and how communities perceive such help. We have contacted you because you have connections to a country where female circumcision is a tradition.”

The interviews were designed as flexible conversations around certain topics, starting with the informants’ family backgrounds, childhood environments, education, whether FGM/C was common where they grew up, and their first awareness of the practice, followed by questions about their lives in Norway and their eventual exposure to FGM/C issues. They were also asked about personal experiences, including their exposure to awareness programs and health services. Finally, informants were asked about defibulation surgeries and their views and experiences regarding these surgeries.

To grasp the informants’ emic perceptions, the interviewer(s) initially made no concrete references to potentially relevant factors. However, when informants mentioned specific factors, such as virility or sexual pleasure, the interviewer(s) probed these topics further. Notably, informants did not have to be asked about their own—or their wives’—FGM/C status, as this information was always freely provided.

The Norwegian Social Science Data Services (NSD) granted ethical approval for this study. The Directorate of Integration and Diversity (IMDi) granted specific clearance to access the quota refugees. The study followed approved ethical procedures, including informed consent in relevant languages. To ensure anonymity while providing a sufficiently thick description, details regarding the informants were kept to a minimum. A few informants were provided with pseudonyms to facilitate reading.

In qualitative research, the researcher is the main methodological tool, and gaining trust is a key task. In interviews with migrants, being an outsider to the community can have both advantages and disadvantages. It can reduce fear of gossip and judgement if the informants were to reveal experiences and considerations that clash with socio-cultural norms within their communities [51]. However, the lack of shared language and experiences may reduce mutual understanding of subtleties. Furthermore, the researcher’s position as a member of the majority population that condemns FGM/C may reduce trust and willingness to share sensitive information.

In this study, trust may have been facilitated through the informants’ perceptions of the researcher as someone in between an insider and an outsider. Despite being an “ethnic Norwegian”, I have travelled and lived in Africa for many years, including Sudan and Somalia, and I have studied FGM/C for almost 20 years. However, what appeared most significant was when informants learned about my former marriage to a Tanzanian, to which many informants exclaimed with apparent relief, “Oh, so you are my sister”. Furthermore, I have worked with and socialized among African diaspora communities in Norway since the early 1980s, and I have numerous lasting relationships with people from the affected communities.

The interpreter who assisted in interviews with the newly arrived Somali refugees was carefully selected, and her role was cautiously chosen to facilitate trust and confidence. She was a mother and had extensive training and experience in social anthropology and social work. To reduce the risk of distrust due to political conflicts based on clan or region, the interpreter was from the same region as the informants. She was probably regarded as an insider because she spoke fluent Somali and shared the FGM/C tradition. At the same time, her Western clothing, mastery of the Norwegian language, and education could have marked her as an outsider. To facilitate the flow of communication, she worked as a co-interviewer rather than an interpreter. Her warmth, sense of humor and relaxed demeanor seemed to put the informants at ease and facilitated their trust.

A final measure to reduce discomfort and fear of repercussions involved avoiding tape-recording the interviews. Instead, detailed notes were taken during the interviews and were subsequently transcribed. Additionally, FGM/C may be a less sensitive topic among the Somali and Sudanese populations than outsiders often expect [18, 52, 53]. In general, most informants spoke freely and answered all queries.

Data analysis was conducted consecutively and at the end of the data collection when the compiled data were reread repeatedly before systematically analyzed by identifying recurrent themes and patterns, as well as exceptions, through a thematic analysis [54]. This analysis included both manual and electronic coding procedures through the use of HyperResearch [55].

## Results

Despite almost uniform resistance to infibulation, a widespread resistance to medicalized defibulation was found in the context of marriage and childbirth. This resistance centered on two major concerns. First, penile defibulation was considered important for men to prove their virility and masculinity; second, full defibulation threatened to create a large vaginal orifice that was regarded as an obstacle to male sexual pleasure.

### Medicalized defibulation may threaten husbands’ virility and masculinity

Both women and men associated penile defibulation with long-term pain and suffering. Additionally, almost all informants knew about the availability of medicalized defibulation. However, when they married, most couples relied on male penetration rather than surgical defibulation. Medical doctors confirmed this impression, with some indicating that only about half or a third of the women who approached the clinics contemplating defibulation actually went through with the surgery. When asked directly about why they resorted to penile defibulation rather than medicalized defibulation, many seemingly had not contemplated their reasons for choosing the former. Most described male defibulation as the normal and acceptable way of ensuring an opening for sexual intercourse, downplaying the pain and suffering involved, while emphasizing penile defibulation as a means of proving men’s virility and masculinity.

Reporting on their marital defibulation, two women described about a month of repeated penile pressure, resulting in open wounds and extreme pain before vaginal intercourse was possible. One, a Sudanese woman in her late 30s, had migrated to Norway 12 years prior to marry. Her way of discussing her type of FGM/C and the opening experience was typical. Initially, she claimed to have “sunna”, which she described as *“removing the tip of the clitoris*”. She also claimed that her first experience of sexual intercourse was unproblematic. However, when she went into detail, both her FGM/C and opening procedure were clearly more extensive than she initially formulated. She continued*, “I had too small opening, so intercourse was painful. It took about a month before we managed. We tried bit by bit. We bought something from the pharmacy, a sort of painkiller gel, but I felt it only made it worse*”. Still, she said that they did not consider surgical defibulation, as “*It wasn’t so bad”.*

Many women described their experience of penile penetration as “not so bad”. They often compared it to horror stories of other women who they knew or had heard about. However, they did describe weeks and months of penile pressure that tore open infibulated scars; women’s screams and cries of pain were considered a normal part of the procedure. Unless specifically asked, the informants rarely mentioned the pain because they seemingly considered it self-evident. Their painful experiences further stressed the need for an exploration of their motivations for resorting to penile penetration, as much of this pain could be avoided through medicalized defibulation.

In several cases, one partner—most often the man but sometimes the woman—resisted medicalized defibulation. A Sudanese woman, approximately 50 years old, mentioned that she had argued with her husband for a long period before he agreed that she could undergo medicalized defibulation when they married in Sudan. He eventually agreed when she promised that she would keep the procedure a secret. Reflecting on the relationship between personal convictions and social norms, she was unsure about what had actually been at stake for her husband:

*“My husband pushed on. He did not want me to have an opening operation. He said he felt pressure from his friends that he had to prove that he could make it. And this, all while he presented himself to me as a modern man who did not want to pressure me. It was just his group of friends who made him to feel pressured. But I felt there was something more there, that it was also an issue for him, that he felt he had to make it. A part of his manhood*”.

All Sudanese informants asserted that medicalized defibulation would be shameful. They told several stories of cases in which couples had suffered and struggled for months without resorting to medicalized defibulation, some of them resorting to risky measures with tools that could seriously harm the woman. Furthermore, the few cases of clinical defibulation were performed in utter secrecy to avoid the shame of failing to create a penile opening. The ways in which the stories were told suggested that many women and men were ambivalent about medicalized defibulation. They discussed penile defibulation not only as a negative practice and painful experience for both women and men but also as a positive way of proving virility and manhood. *“You have to be a man to open the lady”,* a Sudanese man in his late 30s said, priding himself on his accomplishment.

More than one of the informants had been unable to engage in vaginal sexual intercourse for months or even years after their marriage, which clinicians confirmed. One surgeon reported treating a woman after twelve years of marriage. The couple, who had sought help for infertility, had never had vaginal intercourse, and the woman was still fully infibulated.

Another story, told by Omar, a Sudanese man in his 40s, illustrates the ways in which change and mobility can make defibulation an even greater challenge. Omar met and fell in love with his future wife while visiting Sudan, and he brought her to Norway to marry. After six years of marriage, the couple had never had sexual intercourse. Omar said that he had failed to penetrate his wife, as he did not want to use force and inflict pain on her for fear of ruining their relationship: “*If I forced myself on her, she would have suffered. And this pain would be in her mind every time we had sex*”. However, his wife refused to undergo medicalized defibulation, and they eventually divorced. The entire experience “ruined his life”. He was exposed to ridicule and shame by his ex-wife’s family for failing his test of virility and masculinity, as his ex-wife was still a virgin after six years of marriage.

While these ideals of penile penetration—as proof of manhood and virility—were often discussed as a thing of the past or as a custom in countries of origin, they were clearly still valued by many informants, particularly Sudanese men. In contrast, the Somali men and women never emphasized the importance of proving virility through penile penetration in their personal lives. Instead, many women complained about male values of penetration, and two Somali women said their husbands had expressed relief when they told them that they had a less extensive infibulation, thereby reducing defibulation difficulties.

### Tightness and male sexual pleasure

The significance of infibulation persists beyond the test of a man’s virility in the marriage bed; resistance remains regarding the more extensive defibulation necessary for childbirth. At this stage, the extent of defibulation is the issue. Medical guidelines advise that defibulation at the time of marriage be sufficiently large to uncover the urethra in preparation for eventual childbirth. In practice, women enter the delivery room with various degrees of infibulation and defibulation. Some women have undergone partial penile defibulation, while others have requested only partial medicalized defibulation. Some have not been defibulated at all, although this paper does not address such cases. However, when female informants had only partial openings or refused full defibulation during childbirth, they expressed that retaining a small vaginal opening was important because they considered it a prerequisite for male sexual pleasure. Without a tight vaginal orifice, women feared they would be unable to fulfill their husband’s sexual needs, which they feared in turn would tempt men to seek sexual pleasure elsewhere and thereby endanger the marriage. Asha, a Somali woman in her mid-30s explained as follows:

*“All men want tight women. We are so scared that if we are not tight enough, the man will find a new woman to marry or take a younger lover. So, they do some reinfibulation in Somalia also. It is important that the vagina is not a gaping hole. It has to be tight for the man. I feel it myself as well, when we have sex, and if I am very wet, I feel nothing. And my husband says also some times, as a compliment, you were tight today*.”

Many male and female informants shared similar views regarding vaginal tightness as a prerequisite for male sexual pleasure, which was intimately linked to infibulation. A major concern was that childbirth would result in a gaping vaginal opening that was unable to provide male sexual satisfaction. Therefore, many considered reinfibulation to be necessary after childbirth. Almost all the Sudanese men, including those who adamantly opposed infibulation, agreed. Their view is thus in line with the post-partum reinfibulation practiced in Sudan. Furthermore, although reinfibulation is forbidden in Norway, three of the four Sudanese women who had given birth there had experienced pressure to undergo reinfibulation. Only one of them was able to resist the pressure, which was the Sudanese woman who had not undergone any form of FGM/C.

The two other women returned to Sudan for the reinfibulation procedure. Afaf’s husband heavily pressured her to undergo reinfibulation after the birth of their first child in Norway. Her husband sought support from her family to encourage her to undergo reinfibulation, which Afaf found inappropriate and extremely embarrassing. Her reinfibulation resulted in complications and several weeks of suffering. Due to infections, her reinfibulation never healed. Afaf regarded the suffering caused by her reinfibulation as the beginning of the end of her marriage.

Somali informants did not consider reinfibulation a common practice in their country of origin, and none of the Somali women had considered undergoing reinfibulation or had been pressured to do so. By contrast, they enjoyed the ease of bodily functions after marriage and (partial) defibulation. Although Asha indicated that some form of reinfibulation was practiced, she was the only Somali woman who did so, and she provided no details about it; most others insisted that reinfibulation was unheard of. Instead, Somali informants described reclosure as a part of the natural healing process after delivery—often during the 40 days of prescribed post-partum rest.

While both Somali and Sudanese informants valued vaginal tightness as necessary for male sexual pleasure and thus marital stability, its connection to infibulation was unclear. Whereas a vaginal seal could ensure a tight introitus, it would not affect the size or the muscular tightness of the vagina. During infibulation and reinfibulation, tissue from the labia, mostly the labia majora, is stitched together, while the vagina itself is left untouched.

A few informants expressed doubt regarding whether a man could experience sexual pleasure with a woman who was “wide open”, and they thought that reinfibulation was necessary for mothers and previously infibulated women. To explain his support for reinfibulation despite his negative attitudes toward infibulation, a Sudanese man claimed that infibulated women had to be reinfibulated because the original procedure had destroyed vaginal elasticity, resulting in a post-partum vaginal opening that was too large to provide the vaginal tightness necessary for men’s sexual satisfaction. One reason for this perception might be common misconceptions about women’s genitalia, particularly the general lack of awareness of the existence of the urethra as a separate opening from the vaginal introitus [18]. These misunderstandings stunned many health care providers.

Although most public servants were aware of the sexual significance of infibulation, strangely, none of them addressed these topics when working in affected communities. For example, one informant was a school nurse who had run numerous discussion groups on FGM/C for youth on sexuality. When asked whether sexual concerns and the motivation for FGM/C were topic for reflection and discussion in her groups, she was surprised by her own omission. She simply had not considered these topics. Her focus had been on the law and the health risks associated with FGM/C.

## Discussion

A previous paper based on the same data-material found that premarital defibulation is negatively perceived because it is seen to undermine the safeguarding and evidence of virginity that infibulation ensures [4, 17]. However, when women marry and give birth, defibulation is necessary, and clinical procedures would not threaten these core cultural values of virginity and virtue. However, this study found that, also in these contexts, the medicalization of defibulation was commonly resisted. At the time of marriage, medicalized defibulation was considered a threat, undermining men’s attempts to prove their virility and manhood through penile penetration. However, medicalized defibulation at any other time, including childbirth, was also considered a threat due to the extent of the procedure. The larger vaginal orifice often created through medicalized defibulation was seen to jeopardize the tight vaginal introitus regarded as essential for male sexual pleasure. The study thus found that traditional cultural values related to virility and male sexual pleasure remain strong, thereby obstructing the uptake of medicalized defibulation and thus health-seeking behavior.

Some researchers have suggested that the uptake of medicalized defibulation may indicate a changing attitudes toward FGM/C [10, 13]. That is, if people accept clinical defibulation, they not only accept the medicalization of a traditional procedure but this would also suggest that the cultural underpinnings of the practice are losing traction. This assumption actually formed the original idea for this study—to explore whether the uptake of medicalized defibulation could function as a lever of change. While this function is a potential benefit from medicalized defibulation offerings, the study revealed that the cultural values associated with infibulation formed barriers to health care. These very same values could therefore also constitute a barrier to the abandonment of the practice itself.

The informants did not speak with one voice, as several individuals challenged these traditional values. Interestingly, no systematic variation in these attitudes was found with regards to age, age at arrival or time lived in Norway. The only significant variable concerned Sudanese versus Somali informants; the Sudanese emphasizes the values associated with virility and tightness for sexual pleasure more than Somali informants. In contrast to the Sudanese emphasis on proving virility through penile defibulation, more Somali informants sought surgical defibulation upon marrying. This was rarely presented as the result of failed manhood; instead, this choice was associated with care for the woman’s well-being.

What do these complex attitudes and practices indicate about processes of change? In particular, what does the relationship between personal experiences and opinions and the social norms regarding infibulation and its underlying values reveal? To broaden the discussion, I will include findings from a part of the study that examined premarital defibulation [17]. As mentioned, this part of the study identified a strong resistance to premarital defibulation as a perceived threat to values related to women’s virginity and virtue. As such, infibulation seemingly maintain strong symbolic value, which is intimately linked to the physical extent of the procedure. How, then, can it be abandoned?

As outlined above, much work and research on FGM/C over the past decade has focused on perceptions of FGM/C as a social convention and norm. This line of investigation draws heavily upon the work of Garry Mackie [19], whose main theory can be summarized as follows. FGM/C, particularly infibulation, was introduced in what is currently northern Sudan in an attempt to ensure paternity in a highly unequal and hierarchal society. Women of all social strata sought to marry high-ranking men. These men had many wives, which made ensuring their fatherhood. Consequently, families started infibulating their daughters to make them attractive as marriage partners to wealthy men who could provide for them and their children. Over time, the practice of infibulation became the norm, despite the associated pain and health risks. Mackie suggests that this normalization eventually led people to “draw the false interference that women must be excessively wanton to require such scrupulous guarding of their honor” [19] (Op. cit. pp. 263).

Thus, “sexuality”—with regard to ensuring paternity and controlling excess female sexual urges—was seen as central to the institutionalization of FGM/C. However, these sexual concerns do not carry over to theories regarding social conventions, social norms and change. Instead, the emphasis shifts to marriageability, although as a social convention rather than a moral concern. Mackie theorizes that, to be married, women must undergo FGM/C because doing so is the norm; all women follow suit. To abandon FGM/C, a sufficiently large group must agree to stop the practice. In such circumstances, men would accept “uncut” women as marriage partners, and parents would refrain from FGM/C, as they would no longer fear that their uncut daughters were unmarriageable.

This analysis lacks a discussion of how the associations between FGM/C and sexual morality can be loosened. However, Mackie suggests that change will be slower and more difficult in communities where FGM/C is strongly connected to the modesty code, which we found in both the Sudanese and Somali communities in Norway. Furthermore, we observed how the connection between FGM/C and sexuality extends beyond virtue, encompassing values related to manhood and men’s roles and significance. Even in diasporic communities, men must prove their virility and secure their sexual pleasure, even if doing so comes at a high cost for women. Interestingly, values concerning vaginal tightness to ensure male sexual pleasure are not limited to communities practicing infibulation, but found both southern Africa, Asia and western countries [56, 57]. Interestingly, one of the Somali informants claimed that some Somali women in Norway sought vaginal tightening surgeries at private clinics offering so-called genital cosmetic surgery.

Thus, the theories of social convention that inspire many current interventions and ample research seemingly do not capture the sociocultural values upon which the practice hinges. FGM/C is more than a social convention; it encompasses key cultural and personal values related to sexuality and gender roles and relationships. How, then, can it change?

A former study of Somalis in Norway suggested that their changing views on FGM/C was partly fueled by an increased intimacy and interdependence between spouses in Norway that spilled over into their intimate relations [35]. Similar trends are identified in Sudan [58].

## Conclusion

This research found that the factors influencing peoples attitudes, practices, experiences and perceptions are influenced by a multiplicy of factors, including social norms and cultural values, as well as laws, political opinion and personal relations and emotions.

Regarding the social norms and cultural values, this study found that, while most Sudanese and Somali migrants have negative attitudes toward infibulation due to the health risks associated with the practice, they still resist surgical defibulation because it is seen to challenge the cultural values that underlie the practice. These values—women’s virginity and virtue and men’s virility and sexual pleasure—are intimately linked not only to the symbolic value of infibulation but also to the physical extent of the procedure.

As these values remain strong, they limit the acceptance of medicalized defibulation and thus serve as barriers to health-seeking behaviors in response to complications resulting from infibulation. Thus, to ensure adequate health care for girls and women with FGM/C, these cultural values must be addressed.

Furthermore, the same values can also hinder the abandonment of this practice. The most common arguments used to promote health care for those with FGM/C and abandonment of the practice for future generations—the health risks of FGM/C and the health benefits of defibulation—are found to be insufficient to overcome these impediments to change.

Thus, this study suggests that sexual concerns, including the ideals surrounding women’s virginity and morality and men’s virility and pleasure, must be targeted in both medical counselling and preventive interventions. As sexual concerns are a key factor in decisions regarding the continuation or abandonment of FGM/C and the uptake of health services, these issues must be addressed to a significantly higher degree than what is seemingly the case at present.

Such work is also important given the current trend of change in Somalia and Sudan, which often focus on changing the type of FGM/C rather than abandoning the practice entirely. In both countries, negative attitudes toward infibulation are on the rise, accompanied with a growing support for so-called “sunna”. However, as this and several other studies have found, this change is more often observed on a rhetorical, rather than a practical level, as the extent of FGM/C is not always reduced, even if it is described as such [29, 30]. It is worth exploring whether the sexual concerns addressed here also explain why total abandonment of all forms remains difficult and why the strategy of replacing infibulation with “sunna” seems equally difficult. If underlying cultural values do not change, the practice can remain unchanged under another name.

## Abbreviation

FGM/C: Female Genital Mutilation/Cutting
NKVTS: Norwegian Center for Violence and Traumatic Stress Studies
WHO: World Health Organization
